# Structural and Biochemical Analysis of the Recombination Mediator Protein RecR from *Campylobacter jejuni*

**DOI:** 10.3390/ijms241612947

**Published:** 2023-08-18

**Authors:** Su-jin Lee, Si Yeon Ahn, Han Byeol Oh, Seung Yeon Kim, Wan Seok Song, Sung-il Yoon

**Affiliations:** 1 Division of Biomedical Convergence, College of Biomedical Science, Kangwon National University, Chuncheon 24341, Republic of Korea; 2 Institute of Bioscience and Biotechnology, Kangwon National University, Chuncheon 24341, Republic of Korea

**Keywords:** RecR, RecO, DNA, *Campylobacter jejuni*, homologous recombination

## Abstract

The recombination mediator complex RecFOR, consisting of the RecF, RecO, and RecR proteins, is needed to initiate homologous recombination in bacteria by positioning the recombinase protein RecA on damaged DNA. Bacteria from the phylum Campylobacterota, such as the pathogen *Campylobacter jejuni*, lack the *recF* gene and trigger homologous recombination using only RecR and RecO. To elucidate the functional properties of *C. jejuni* RecR (cjRecR) in recombination initiation that differ from or are similar to those in RecF-expressing bacteria, we determined the crystal structure of cjRecR and performed structure-based binding analyses. cjRecR forms a rectangular ring-like tetrameric structure and coordinates a zinc ion using four cysteine residues, as observed for RecR proteins from RecF-expressing bacteria. However, the loop of RecR that has been shown to recognize RecO and RecF in RecF-expressing bacteria is substantially shorter in cjRecR as a canonical feature of Campylobacterota RecR proteins, indicating that cjRecR lost a part of the loop in evolution due to the lack of RecF and has a low RecO-binding affinity. Furthermore, cjRecR features a larger positive patch and exhibits substantially higher ssDNA-binding affinity than RecR from RecF-expressing bacteria. Our study provides a framework for a deeper understanding of the RecOR-mediated recombination pathway.

## 1. Introduction

All living organisms recognize and repair DNA damage, such as dsDNA breaks and ssDNA gaps, via homologous recombination, thus maintaining the integrity of their genomes [1]. Homologous recombination requires a search of homologous DNA, which is mediated by the recombinase protein RecA in bacteria. RecA helically coats ssDNA in damaged DNA and induces the formation of a synaptic complex between homologous DNA strands. RecA loading on ssDNA is initiated by the recognition of DNA damage by a recombination mediator complex. One of the critical recombination mediator complexes is RecFOR, which consists of the RecF, RecO, and RecR proteins. The RecFOR complex detects and binds the ssDNA–dsDNA junction at one end of a ssDNA gap that is coated with ssDNA-binding proteins (SSB) [2,3]. Subsequently, RecFOR locates RecA on ssDNA in place of SSB.

A recent structural study of a complex between RecFOR and dsDNA with a ssDNA extension provided a mechanism for the cooperative binding of RecFOR to gapped DNA [3]. A RecF dimer clamps dsDNA and simultaneously interacts with RecR, facilitating the positioning of a RecR tetramer in the dsDNA–ssDNA junction. As a result, RecR directs RecO to the ssDNA side through specific binding to RecO and is expected to recruit RecA on SSB-coated ssDNA [3,4]. However, this model is difficult to generalize to all bacterial species, given that some bacterial species, including *Campylobacter jejuni* and *Helicobacter pylori*, do not have the *recF* gene and employ the RecF-independent RecOR pathway for homologous recombination [5,6]. Moreover, the RecOR pathway has been shown to coexist with the RecFOR pathway even in RecF-expressing bacteria [7]. However, the exact mechanism whereby RecOR alone recognizes gapped DNA without RecF is unclear. 

Given that RecR interacts with RecF or RecO for the initiation of homologous recombination, RecR functions as the most critical recombination mediator protein in both the RecFOR and RecOR pathways [3,8,9]. Consistently, RecR is the most conserved protein with respect to sequence among RecF, RecO, and RecR. Based on crystal structures, RecR alone assembles into a ring-shaped tetramer [4,8,10,11,12]. A RecR tetramer forms a heterocomplex with one or two RecO polypeptide chains and displays binding affinity for DNA when RecO coexists [3,4,8,9,12]. However, these features have been revealed in RecF-expressing bacteria from the phyla Deinococcota, Actinomycetota, and Pseudomonadota and have not been characterized in RecF-deficient bacteria. RecR from RecF-deficient bacteria is expected to display both common and unique features compared to that from RecF-expressing bacteria, as shown for RecO [6,13]. RecO from RecF-deficient *Campylobacter jejuni*, which belongs to the phylum Campylobacterota and causes food poisoning in humans, presents a curved three-domain structure, as shown for other RecO orthologs [6]. However, *C. jejuni* RecO (cjRecO) coordinates a zinc ion using a unique sequence and has substantially lower ssDNA-binding affinity than SSB and other RecO proteins. 

To identify the structural and biophysicochemical features of RecR that are conserved in all bacterial species or unique to RecF-deficient bacteria regarding the initiation of homologous recombination, we analyzed the crystal structure of *C. jejuni* RecR (cjRecR) and its zinc ion-, DNA-, and RecO-binding capacities. As observed in other RecR proteins, cjRecR forms a tetramer and harbors a zinc ion for structural stability. However, cjRecR displayed unique interactions with DNA and RecO. Our study of the unique cjRecR protein will contribute to the development of *C. jejuni*-targeted antibiotics, given that humans who consume undercooked poultry contaminated with *C. jejuni* develop enteritis with fever and diarrhea, which can lead to Guillain–Barre syndrome characterized by acute paralysis [14,15,16]. 

## 2. Results and Discussion

### 2.1. Overall Structure of cjRecR

**Figure 1:**
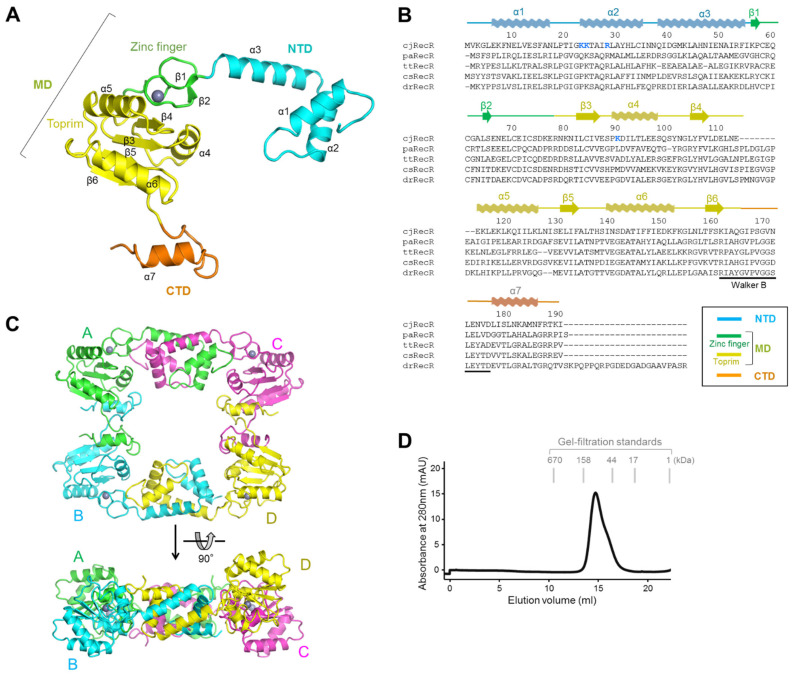
Overall structure of cjRecR and its tetramerization. (**A**) Overall structure of a cjRecR monomer. The cjRecR structure is shown as ribbons, which are differently colored for the NTD (cyan), zinc finger subdomain (green), Toprim subdomain (yellow), and CTD (orange). (**B**) Sequence alignment of cjRecR and its orthologs. The secondary structures of cjRecR are represented by waves (α-helices), arrows (β-strands), and lines (coils) in domain- or subdomain-specific colors above the sequence. The positively charged residues of the cjRecR central pore (K23, K24, R28, and K90) are shown in bold blue fonts. (**C**) Tetrameric structure of cjRecR. The four cjRecR monomers are shown as differently colored ribbons (Chain A, green; Chain B, cyan; Chain C, magenta; Chain D, yellow). (**D**) Gel-filtration chromatography analysis of cjRecR.

The crystal structure of cjRecR was obtained at a 2.6 Å resolution using the recombinantly expressed cjRecR protein (residues 1–190) (Table S1). The asymmetric unit of cjRecR contains four cjRecR subunits. The structure of a cjRecR monomer (residues 1–189) consists of seven α-helices (α1–α7) and six β-strands (β1–β6) and is spatially separated into three parts, including the N-terminal domain (NTD), middle domain (MD), and C-terminal domain (CTD) (Figure 1A,B). The NTD, MD, and CTD are serially arranged into a shape consisting of two arms at a right angle, as observed for other RecR orthologs from RecF-expressing bacteria, including *Thermus thermophilus* RecR (ttRecR), *Caldanaerobacter subterraneus* RecR (csRecR), *Pseudomonas aeruginosa* RecR (paRecR), and *Deinococcus radiodurans* RecR (drRecR) (Figure S1A) [10,11,12,17]. The MD is located in the middle of the cjRecR monomer structure, similar to an elbow, and the NTD and CTD extend from the MD like the upper and lower arms, respectively (Figure 1A,B). The NTD (residues 1–55) contains three N-terminal α-helices (α1–α3). The MD (residues 56–164) consists of zinc finger and Toprim subdomains, which are closely packed into one domain. The zinc finger subdomain (residues 56–77) contains two short β-strands (β1 and β2) and accommodates a zinc ion. The Toprim subdomain (residues 78–164) exhibits a four-stranded β-sheet that is decorated by α-helices (α4–α6) on both sides. The CTD (residues 165–189) is a small domain with a C-terminal α-helix (α7) and harbors the Walker B motif at the β6–α7 loop.

The four cjRecR subunits from the asymmetric unit exhibit essentially identical structures for each domain, with root-mean-square deviation (RMSD) values of 0.18–0.48 Å (Figure S2). However, when the full-length structures are compared, the RMSD value increases to over 1 Å because of differences in interdomain angles, in particular for Chain D (RMSD values between Chain D and other chains, ~2.6 Å), indicating the interdomain flexibility of cjRecR. 

### 2.2. Tetrameric Structure of cjRecR

The four cjRecR chains from the asymmetric unit assemble into a tetramer in the shape of a rectangular ring with a central pore, as observed for other RecR structures (Figure 1C and Figure S1B) [10,11,12,17]. The central pore comprises residues from the α1–α2 loop, α2, and α4 helices, and β6–α7 loop (Figure 1C). Each vertex of the rectangular tetramer is occupied by one MD. The α5 and α6 helices that decorate one face of the central β-sheet in the MD Toprim subdomain protrude slightly from the rectangular plane of the cjRecR tetramer. The Toprim subdomain from Chain D protrudes more than those of other chains because of the irregular interdomain angle of Chain D.

cjRecR tetramerization is mainly mediated by NTD-NTD′ homodimerization and MD-CTD′ heterodimerization (the prime denotes another polypeptide chain) (Figure 1C). The dimerization interface is extensive. The NTD of Chain A (or Chain B) forms a homodimer with that of Chain C (or Chain D) at one side of the cjRecR rectangle by engaging all the secondary structures with a buried surface area of ~2100 Å^2^. On another side of the cjRecR rectangle, the CTD from Chain A (or Chain C) interacts with one face of the Toprim subdomain of Chain B (or Chain D) using all the secondary structures with a buried surface area of ~2000 Å^2^. 

The tetrameric assembly of cjRecR was confirmed using gel-filtration chromatography (Figure 1D). In gel-filtration chromatography, the cjRecR protein was primarily eluted between the 44 and 158 kDa standards, and its apparent molecular size was estimated to be ~90.5 kDa, which is close to the calculated molecular weight of one cjRecR tetramer (~88.3 kDa). Notably, a shoulder peak is observed on the right side of the tetramer peak and seems to correspond to a dimer. These observations indicate that cjRecR primarily forms a tetramer both in crystal and in solution with additional low levels of dimer in solution. The high propensity to form a tetramer in solution is unique to cjRecR, given that ttRecR, csRecR, paRecR, and drRecR have been shown to exist as dimers in solution, in contrast to their crystal structures [8,10,11,12]. 

### 2.3. Zinc-Binding Motif of cjRecR and Its Critical Role in Protein Stability

**Figure 2:**
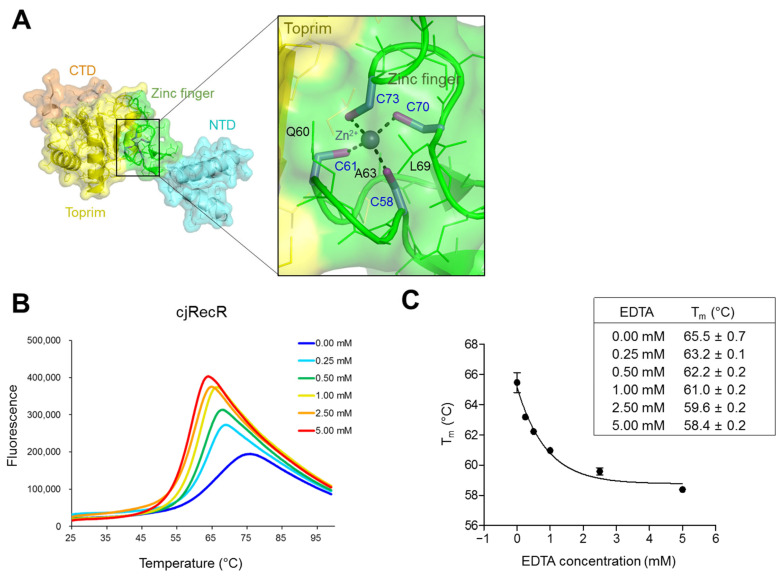
Zinc ion coordination by cjRecR and its critical role in protein stability. (**A**) Zinc ion coordination by the zinc finger motif of cjRecR. (**B**,**C**) Critical role of a zinc ion in the protein stability of cjRecR. The T_m_ of cjRecR (**C**) was determined in the presence and absence of EDTA via a thermal shift assay (**B**). Data (**B**) are representative of three independent experiments that yielded similar results, and the T_m_ values (**C**) represent the means ± S.D. from the three independent experiments.

In the cjRecR structure, each cjRecR subunit accommodates one zinc ion using the zinc finger subdomain (Figure 1C and Figure 2A). The zinc ion is simultaneously coordinated by four conserved cysteine residues (C58, C61, C70, and C73) from the two CxxC motifs at the β1–β2 and β2–β3 loops, stabilizing the relatively long β1–β2 and β2–β3 loops, which would otherwise be highly flexible (Figure 1B and Figure 2A). Moreover, the Zn-Cys cluster grabs the Q60 and L69 residues from the β1–β2 and β2–β3 loops, respectively, at its top and interacts with the A63 residue from the β1–β2 loop at its bottom, contributing to the stabilization of the zinc finger subdomain that is prevalent with coil structures (coil, 82%; β-strand, 18%) (Figure 1A,B and Figure 2A). Notably, the β1–β2 and β2–β3 loops of the zinc finger subdomain participate in inter-subdomain interactions with the Toprim subdomain. Therefore, we propose that zinc ion-mediated stabilization extends over the four cysteine residues to the entire MD domain. 

To verify the critical role of the Zn-Cys cluster in the stability of cjRecR, the melting temperature (T_m_) of the cjRecR protein was determined in the absence and presence of the divalent metal ion-chelating reagent EDTA using a thermal shift assay (Figure 2B). The cjRecR protein exhibited a canonical temperature-dependent denaturation curve with a T_m_ of 65.5 °C (Figure 2B,C). Although cjRecR forms a tetramer, only one thermal transition was observed, implying that thermal stress simultaneously disrupts monomer folding and tetramerization (Figure 2B). EDTA decreased the T_m_ value of the cjRecR protein. A rapid decrease in T_m_ was observed with up to 1 mM EDTA, and the T_m_ decrease became small at EDTA concentrations higher than 1 mM. The T_m_ of cjRecR decreased from 65.5 °C to 58.4 °C by 2.5 mM EDTA, indicating that zinc ion abstraction from the cjRecR protein substantially decreases the stability of cjRecR to be consistent with our structural finding that zinc ion-mediated stabilization extends to the whole MD domain (Figure 2B,C and Figure S3). Therefore, we conclude that zinc ion coordination by the cysteine residues is required to maintain the protein stability of cjRecR. 

### 2.4. DNA-Binding Ability of cjRecR

**Figure 3:**
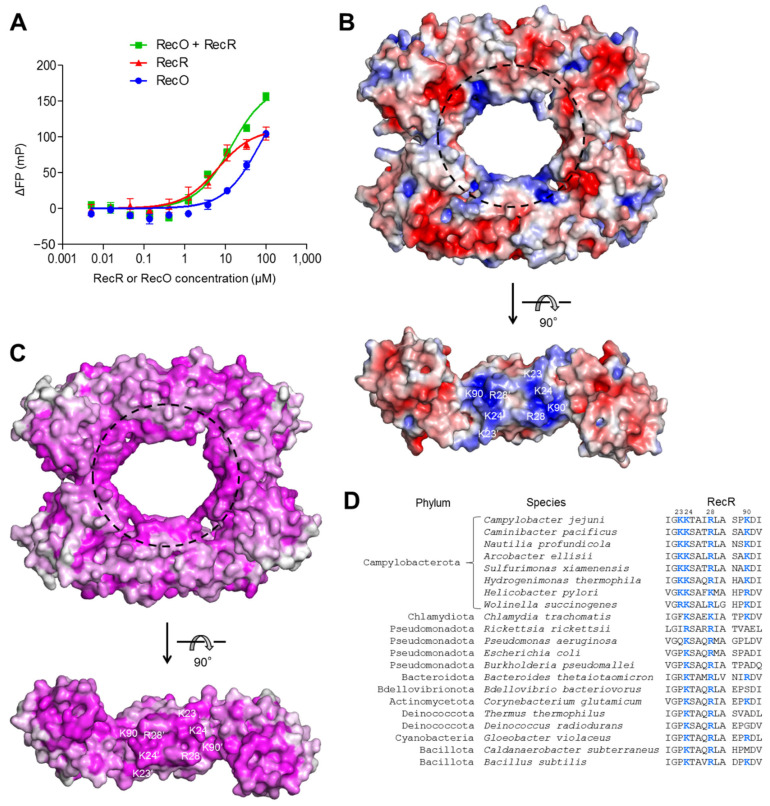
DNA recognition by cjRecR. (**A**) Interaction of cjRecR and cjRecO with ssDNA. The interaction was analyzed via a fluorescence polarization assay by incubating cjRecR, cjRecO, or their mixture with fluorescein-labeled ssDNA and determining fluorescence polarization signals. The K_d_ for the cjRecR–ssDNA interaction is 6.8 ± 2.7 μM (mean ± standard deviation). (**B**) Putative DNA-binding site of cjRecR (dashed circle). The tetrameric structure of cjRecR is shown as electrostatic potential surfaces (positive, blue; neutral, white; negative, red). The positively charged residues of cjRecR at the putative DNA-binding site are indicated in the lower panel. For clarity, the RecR structure is shown in a dimeric form without the CTDs in the lower panel. (**C**) Sequence conservation of the putative DNA-binding site of RecR. The sequence conservation was calculated in the ConSurf server (consurf.tau.ac.il, accessed on 25 May 2023) [18] using RecR ortholog sequences that share over 40% sequence identity with cjRecR and is shown in surface representation in proportion to magenta intensity in an orientation identical to that of Figure 3B. (**D**) Sequences of RecR orthologs from diverse phyla in and near the predicted DNA-binding region. The positively charged residues of the cjRecR central pore (K23, K24, R28, and K90) and their identical residues in orthologs are shown in bold blue fonts. Bacterial species from the phylum Campylobacterota lack the *recF* gene, whereas other species listed in the figure have the *recF* gene.

RecR proteins, including drRecR and csRecR, have been demonstrated to display no significant binding to ssDNA [8,9,12]. However, RecR improved the ssDNA-binding affinity of RecO by forming a complex with RecO. For example, the K_d_ for the interaction between drRecO and ssDNA decreased by ~6-fold from 8.7 μM to 1.4 μM in the presence of drRecR [8]. To address the DNA-binding capacity of *C. jejuni* recombination mediator proteins, a fluorescence polarization assay was performed for cjRecR and cjRecO using fluorescein-labeled 40-mer ssDNA (Figure 3A). Interestingly, unexpected results were obtained for the interactions of cjRecR and cjRecO with ssDNA. First, cjRecR substantially interacted with ssDNA, with a K_d_ value of 6.8 μM, in contrast to drRecR and csRecR. Second, cjRecR exhibited a higher ssDNA-binding affinity than cjRecO, whereas the opposite was observed for the counterparts from *D. radiodurans*. Third, the significant increase in ssDNA-binding affinity achieved by the coexistence of drRecR and drRecO was not observed for cjRecR and cjRecO. When cjRecO coexisted with cjRecR, only an additive ssDNA-binding effect was detected. 

To identify the DNA-binding site of cjRecR, the electrostatic potential surfaces of cjRecR were analyzed using the tetrameric structure (Figure 3B). Positive patches, which are expected to interact with the negatively charged phosphate backbone, are observed on the internal surface of the cjRecR ring structure, whose residues are highly conserved across bacterial species from the Campylobacter phylum (Figure 3B,C). DNA recognition by the central pore of cjRecR is consistent with a previous small-angle neutron scattering analysis of drRecOR and DNA [8]. The positive patch of cjRecR in the central pore includes positively charged residues, K23, K24, R28, and K90, from each cjRecR chain (Figure 3B). Among these four residues, the cjRecR K24 and R28 residues are conserved as positively charged residues (lysine or arginine) across RecR orthologs (Figure 3B–D). The critical role of these two positively charged residues in DNA binding was confirmed in a previous mutational study on drRecR [9,17]. In contrast to the cjRecR K24 and R28 residues, cjRecR K23 and K90 are conserved as positively charged residues in the phylum Campylobacterota and are generally replaced with neutral residues in other bacterial phyla, most species of which, including *C. subterraneus* and *D. radiodurans*, have the *recF* gene, highlighting the unique role of the additional positive residues in DNA recognition by Campylobacterota RecR proteins. Consistently, cjRecR displayed substantial binding to ssDNA, whereas drRecR and csRecR did not (Figure 3A) [8,9,12]. Given that Campylobacterota species do not express RecF, which contributes to DNA recognition, RecR proteins from RecF-deficient Campylobacterota bacteria seem to have evolved the ability to bind DNA more efficiently than RecR proteins from RecF-expressing bacteria [5,6].

### 2.5. Uniquely Short β4–α5 Loop of cjRecR and Its Implication in the Interaction of cjRecR with Other Recombination Mediator Proteins

**Figure 4:**
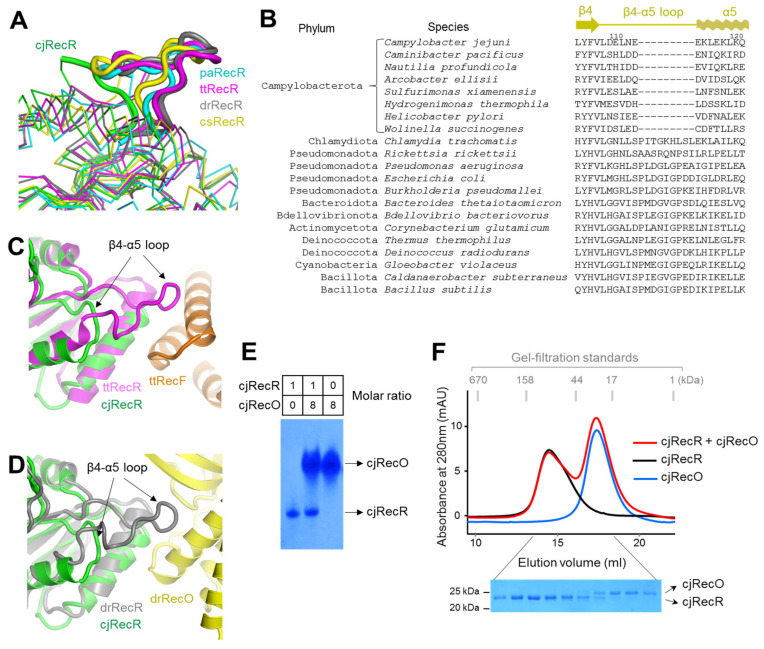
Short β4–α5 loop of cjRecR and its impact on RecO or RecF binding. (**A**) Short β4–α5 loop of cjRecR (green) in comparison with the long β4–α5 loops of paRecR (PDB ID 5Z2V; cyan), ttRecR (PDB ID 5ZVQ; magenta), drRecR (PDB ID 1VDD; gray), and csRecR (PDB ID 3VDP; yellow). The RecR proteins are shown as Cα traces, and their β4–α5 loops are highlighted in thick coils. (**B**) Sequences of RecR orthologs from diverse phyla at the β4–α5 loop. (**C**) Lack of interaction between the cjRecR β4–α5 loop and RecF due to its short length. The cjRecR structure (green ribbons) is superimposed on the ttRecFOR-DNA complex structure (PDB ID 8BPR; ttRecR, magenta ribbons; ttRecF, orange ribbons). (**D**) Lack of interaction between the cjRecR β4–α5 loop and RecO due to its short length. The cjRecR structure (green ribbons) is superimposed on the drRecOR complex structure (PDB ID 4JCV; drRecR, gray ribbons; drRecO, yellow ribbons). (**E**) Native PAGE analysis of cjRecR, cjRecO, and their mixture. (**F**) Gel-filtration chromatography analysis of cjRecR, cjRecO, and their mixture (upper panel) and SDS-PAGE analysis of the gel-filtration chromatography fractions (lower panel).

cjRecR exhibits the largest structural difference in the β4–α5 loop of the Toprim subdomain from the structurally defined RecR orthologs from RecF-expressing bacteria, including ttRecR, csRecR, paRecR, and drRecR (Figure 4A) [10,11,12,17]. In the cjRecR structure, the β4 strand is connected to the α5 helix by six residues at the β4–α5 loop. In the ttRecR, csRecR, paRecR, and drRecR structures, the β4–α5 loop is nine residues longer than that of cjRecR and forms the most prominent region that protrudes from the rectangular plane of the tetramer. The sequence alignment of RecR orthologs indicates that the short β4–α5 loop is found only in RecR proteins from the phylum Campylobacter, whereas RecR proteins from all other bacterial phyla feature a long β4–α5 loop (Figure 4B). Therefore, the short β4–α5 loop is a canonical feature of Campylobacterota RecR proteins not observed in other phyla. Interestingly, the protruding β4–α5 loop of RecR from *T. thermophilus* is required for RecF binding as one of two key RecF-binding regions (Figure 4C) [3]. Given that the genome of *C. jejuni* does not contain the *recF* gene, unlike that of *T. thermophilus*, *C. jejuni* was not under the functional constraint to retain the long β4–α5 loop as a RecF binder and thus lost the nine residues from the β4–α5 loop during the evolution of the *recR* gene.

Based on the drRecOR complex structure, the protruding, long β4–α5 loop of drRecR is also involved in the interaction with RecO (Figure 4D) [8]. Because cjRecR has a short β4–α5 loop, cjRecR is expected to have lower cjRecO-binding affinity than other RecR proteins from RecF-expressing bacteria. Indeed, the interaction of cjRecR with cjRecO was too weak to be detected using native PAGE and gel-filtration chromatography, which analyze relatively strong binding (Figure 4E,F). However, both the csRecOR and drRecOR complexes were identified in solution via gel-filtration chromatography, and drRecR was shown to exist as a 4:2 heterocomplex with drRecO in the crystal structure [8,9,12]. 

In conclusion, cjRecR assembles into a rectangular ring-like tetrameric structure that accommodates zinc ions, as reported for RecR proteins from RecF-expressing bacteria. However, the inner surface of the cjRecR tetrameric ring provides a uniquely extensive positive patch, contributing to cjRecR binding to DNA. Additionally, the cjRecR structure has a unique short β4–α5 loop, indicating that cjRecR has a low cjRecO-binding affinity. Our study of cjRecR will provide a framework to understand the RecF-independent RecOR pathway in bacteria. A further structural study of DNA recognition by the RecOR complex is required to determine the exact mechanism by which RecOR mediates recombination in the absence of RecF. 

## 3. Materials and Methods

### 3.1. Construction of the Protein-Expression Plasmid

The cjRecR protein-expression plasmid was constructed by PCR, restriction enzyme-mediated digestion, and ligation. The DNA fragment that encodes the cjRecR protein was generated by PCR using the genomic DNA of *C. jejuni* subsp. *jejuni* (ATCC 33291) as a DNA template with DNA primers containing the recognition sequence of the BamHI or SalI restriction enzyme at one end. The PCR product was treated with the BamHI and SalI restriction enzymes and was ligated using T4 DNA ligase into a pET49b plasmid variant that was designed to express RecR protein in fusion with an N-terminal hexahistidine tag and a subsequent thrombin cleavage sequence [19]. The nucleotide sequence of the insert in the cjRecR expression plasmid was confirmed using DNA sequencing.

### 3.2. Protein Expression and Purification

*E. coli* BL21 (DE3) cells containing the cjRecR-expression plasmid were grown in an LB medium at 37 °C. When the optical density of the culture at 600 nm reached 0.6, the culture was supplemented with isopropyl β-D-1-thiogalactopyranoside (1 mM) to induce cjRecR overexpression. The cells were further cultured at 37 °C for 3 h. The resultant cells were harvested using centrifugation and lysed using sonication in 50 mM Tris, pH 8.0, 200 mM NaCl, and 5 mM β-mercaptoethanol (βME). 

The cjRecR protein was first purified using immobilized metal affinity chromatography. The cell lysate containing the hexahistidine-tagged cjRecR protein was incubated with Ni-NTA resin, and the resin was harvested using an Econo-Column (Bio-Rad, Hercules, CA, USA). The cjRecR protein was eluted from the resin using a solution containing 50–500 mM imidazole, 50 mM Tris, pH 8.0, 200 mM NaCl, and 5 mM βME. The eluted cjRecR protein was dialyzed against 20 mM Tris pH, 8.0, and 5 mM βME and then treated with thrombin to remove the hexahistidine tag. Subsequently, the untagged cjRecR protein was purified via anion exchange chromatography using a Mono Q 10/100 column (GE Healthcare, Chicago, IL, USA) with a NaCl gradient (0–500 mM) in 20 mM Tris, pH 8.0, and 5 mM βME. 

cjRecO protein was produced in *E. coli* cells and purified by affinity and ion exchange chromatography as described previously [6].

### 3.3. Protein Crystallization and X-ray Diffraction

The cjRecR protein was crystallized using a sitting-drop vapor-diffusion method at 18 °C with a reservoir solution containing 17% ethanol, 0.1 M Hepes, pH 7.5, and 0.25 M magnesium chloride. A cjRecR crystal was cryoprotected using 25% ethylene glycol and flash-cooled under a gaseous nitrogen stream at −173 °C. To obtain X-ray diffraction data, X-rays were applied to the cjRecR crystal at the Pohang Accelerator Laboratory (Republic of Korea). These diffraction data were indexed, integrated, and scaled using the HK2000 program [20]. 

### 3.4. Structure Determination

The crystal structure of cjRecR was determined using a molecular replacement method using X-ray diffraction data up to a 2.6 Å resolution. A molecular replacement solution was obtained using the Phaser program with the *P. aeruginosa* RecR structure (PDB ID 5Z2V) as a search model [21]. The resulting cjRecR model was subjected to iterative cycles of manual rebuilding and automatic refinement using the Coot and Phenix.refine programs, respectively, to generate the final tetrameric structure of cjRecR [22,23]. 

### 3.5. Gel-Filtration Chromatography

To determine the oligomeric state of cjRecR by gel-filtration chromatography, the purified cjRecR protein (~300 μg) in 300 μL of an analysis solution (20 mM Tris, pH 8.0, 150 mM NaCl, and 5 mM βME) was loaded onto a Superdex 200 10/300 column and was eluted in the analysis solution. Protein elution levels were determined upon measuring the UV absorbance at 280 nm. To estimate the molecular size of the RecR protein, the elution volume of the cjRecR protein was compared with those of gel-filtration standards (Bio-Rad).

Gel-filtration chromatography was also performed to determine whether cjRecR interacts with cjRecO with a high binding affinity. cjRecR (175 μg), cjRecO (100 μg), and their mixture (175 μg cjRecR and 100 μg cjRecO) at a 2:1 molar ratio in 300 μL of the analysis solution were individually loaded onto a Superdex 200 10/300 column and eluted in the analysis solution. The fractions from gel-filtration chromatography were analyzed using SDS-PAGE.

### 3.6. Thermal Shift Assay

For a thermal shift assay, the purified cjRecR protein (10 μM) was incubated with EDTA at various concentrations (0 mM, 0.25 mM, 0.50 mM, 1.00 mM, 2.50 mM, or 5.00 mM) in the presence of 500-fold diluted SYPRO Orange (Invitrogen catalog number S6651) in a solution containing 20 mM Tris, pH 8.0, and 5 mM βME. The fluorescence intensity of SYPRO Orange (excitation wavelength, 470 ± 15 nm; emission wavelength, 520 ± 15 nm) in the cjRecR protein solution was determined at 25–99 °C using a QuantStudio 1 Real-Time PCR System (Thermo Fisher Scientific). The T_m_ value of cjRecR was calculated using the wTSA-CRAFT web server [24]. The T_m_ value of a control protein (*Xanthomonas campestris* FliD) was also determined in a manner identical to that of the cjRecR protein. 

### 3.7. Fluorescence Polarization Assay

For a fluorescence polarization assay, a protein sample (cjRecR, cjRecO, and their mixture) at various concentrations up to 100 μM was incubated with a fluorescein-probed 40-mer ssDNA (5′-TTATAGGCATATAGGAGTAATTTTCTTGGGCTATGCAGTA-3′; 0.8 nM). The fluorescence polarization level of the protein–ssDNA solution was determined using an Infinite F200 PRO instrument (Tecan, Männedorf, Switzerland). The dissociation equilibrium constant (K_d_) for the cjRecR–ssDNA interaction was estimated using a one-site binding model with the Prism 5 software (GraphPad, San Diego, CA, USA).

### 3.8. Native PAGE

To address the cjRecR-cjRecO interaction, a native PAGE was performed. Protein samples (cjRecR, cjRecO, and their mixture) were incubated at 18 °C for 30 min in 20 mM Tris, pH 8.0, 150 mM NaCl, and 5 mM βME, and native PAGE was then performed using a 6% polyacrylamide gel at 100 V. The resulting gel was stained using Coomassie brilliant blue. 

## Data Availability

The atomic coordinates and the structure factors for cjRecR (PDB ID 8K3F) have been deposited in the Protein Data Bank (www.rcsb.org, deposited on 15 July 2023).

## Supplementary Materials

The supporting information can be downloaded at: https://www.mdpi.com/article/10.3390/ijms241612947/s1.

## Abbreviations

βME: β-mercaptoethanol; cjRecO, *Campylobacter jejuni* RecO; cjRecR, *Campylobacter jejuni* RecR; csRecR, *Caldanaerobacter subterraneus* RecR; CTD, C-terminal domain; drRecR, *Deinococcus radiodurans* RecR; MD, middle domain; NTD, N-terminal domain; paRecR, *Pseudomonas aeruginosa* RecR; RMSD, root-mean-square deviation; SSB, ssDNA-binding protein; T_m_, melting temperature; ttRecR, *Thermus thermophilus* RecR.
