# Supplementary material for: Structural and Biochemical Analysis of the Recombination Mediator Protein RecR from Campylobacter jejuni
Source: Int J Mol Sci. 2023 Aug 18;24(16):12947. doi: 10.3390/ijms241612947 (PMC10454854; doi:10.3390/ijms241612947)
Supplement: Supplementary file 1 [file ijms-24-12947-s001.zip › ijms-2553907-supplementary.pdf]

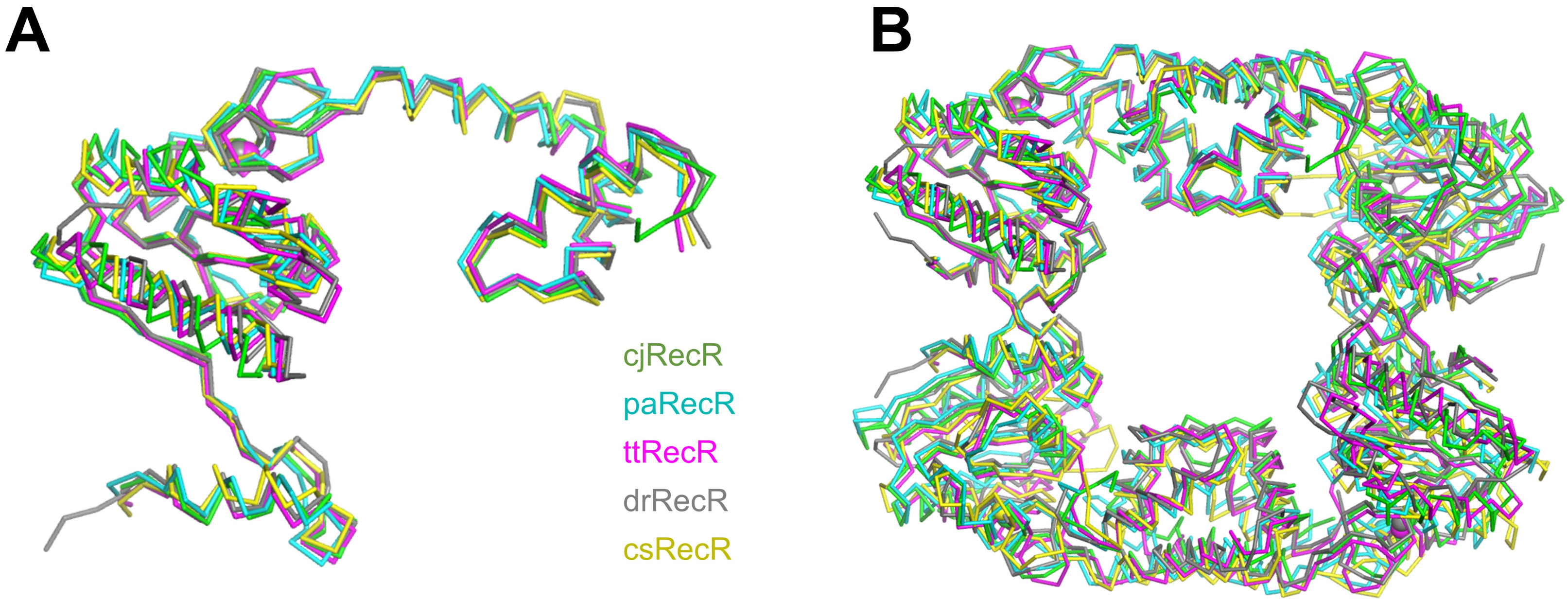

Figure S1. Similar structures of cjRecR (green), paRecR (PDB ID 5Z2V; cyan), ttRecR (PDB ID 5ZVQ; magenta), drRecR (PDB ID 1VDD; gray), and csRecR (PDB ID 3VDP; yellow). RecR proteins and zinc ions are shown as Cα traces and spheres, respectively. (**A**) Similar monomer structures of RecR orthologs. (**B**) Similar tetramer structures of RecR orthologs. The tetrameric structures are overlaid using the monomer structures shown in Fig. S1A.

A

RMSD (Å) between cjRecR chains

	A-B	A-C	A-D
Full-length	0.42	1.08	2.57
NTD	0.37	0.37	0.42
MD	0.19	0.29	0.39
CTD	0.18	0.60	0.48

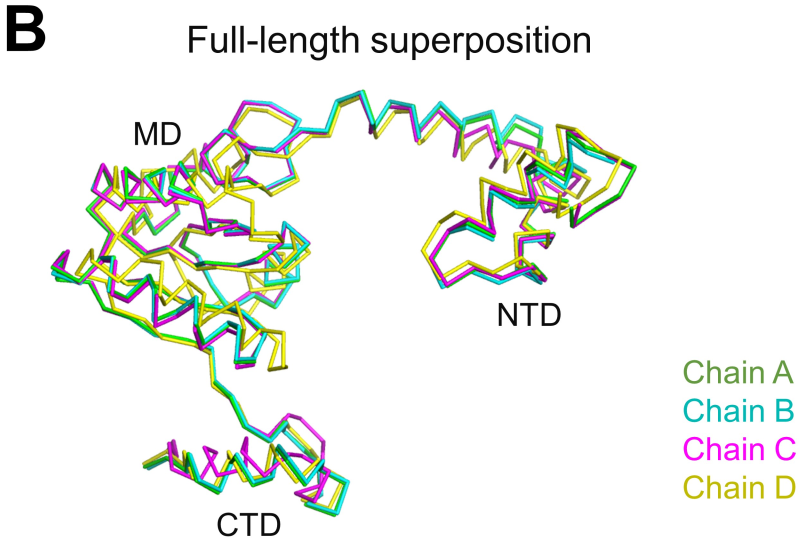

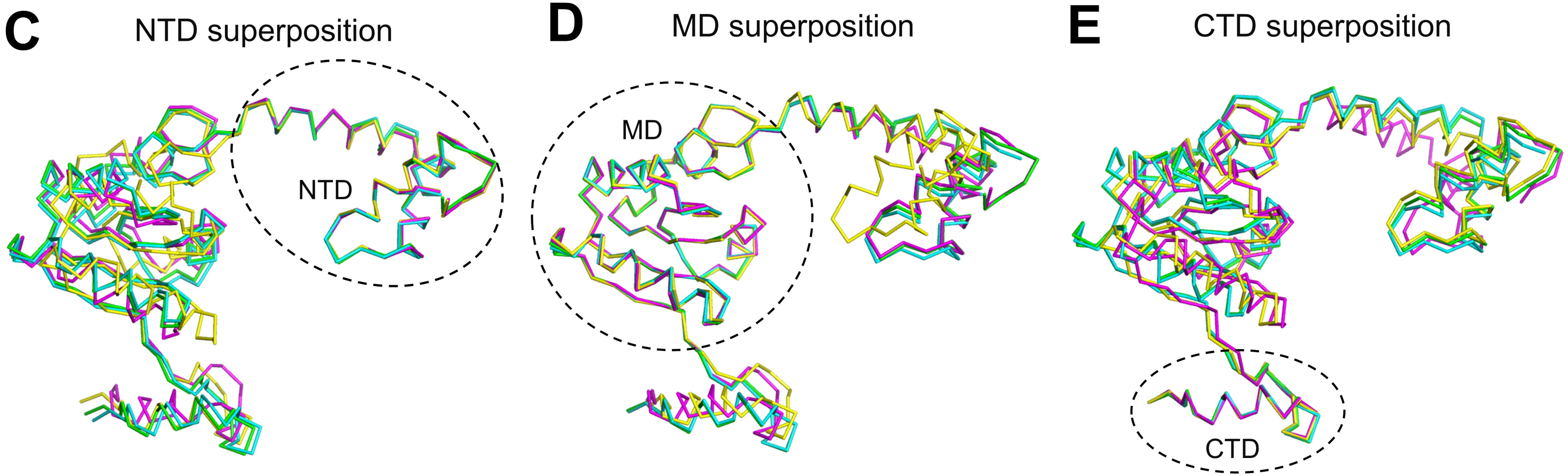

Figure S2. Overlays of the four cjRecR chains observed in the asymmetric unit of the cjRecR crystal. (**A**) RMSD values between the four cjRecR chains for the entire structure or each domain. (**B**) cjRecR chains superimposed using the entire structure. (**C**) cjRecR chains superimposed using the NTD. (**D**) cjRecR chains superimposed using the MD. (**E**) cjRecR chains superimposed using the CTD.

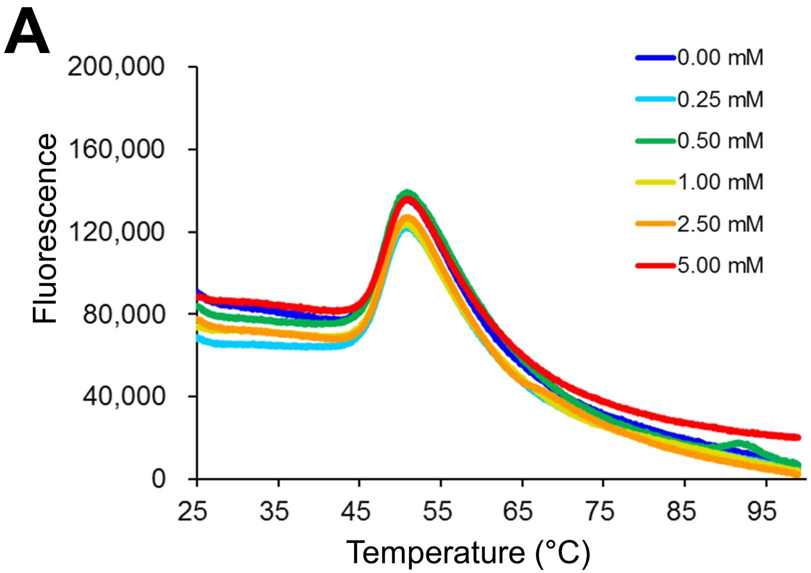

B

EDTA	T_m (°C)
0.00 mM	47.7 ± 0.0
0.25 mM	47.7 ± 0.1
0.50 mM	47.7 ± 0.0
1.00 mM	47.6 ± 0.1
2.50 mM	47.7 ± 0.1
5.00 mM	47.8 ± 0.1

Figure S3. No significant change in the protein stability of the *X. campestris* FliD control protein in response to EDTA. The T_m of FliD (**B**) was determined in the presence and absence of EDTA via a thermal shift assay (**A**). The data (**A**) are representative of three independent experiments that yielded similar results, and the T_m values (**B**) represent the means ± S.D. from the three independent experiments.

Table S1. Crystallographic statistics of the cjRecR structure.

	cjRecR
**Data collection**	
Space group	C2
Cell parameters	a = 231.43, b = 64.19, c = 74.62, β = 100.60°
Wavelength (Å)	0.9793
Resolution (Å)	30.00-2.60
Highest resolution (Å)	2.64-2.60
No. unique reflections	32,226 (1,614) [a]
R_{merge} (%) [b]	7.1 (59.1) [a]
R_{meas} (%) [c]	8.5 (71.0) [a]
R_{pim} (%) [d]	4.7 (38.9) [a]
$CC_{1/2}$ [e]	0.992 (0.666) [a]
I/sigma(I)	19.5 (2.1) [a]
Completeness (%)	97.0 (97.2) [a]
Redundancy	2.8 (2.8) [a]
**Refinement**	
Resolution (Å)	30.00-2.60
No. of reflections (work)	30,605
No. of reflections (test)	1,616
R_{work} (%) [f]	20.7
R_{free} (%) [g]	24.6
No. atoms	
Protein	5,659
Zinc ion	4
Water	32
Average B-value ($Å^2$)	61.3
RMSD bonds (Å)	0.004
RMSD angles (°)	0.630
Ramachandran [h] (favored)	95.3%
(outliers)	0.0%

[a] Numbers in parentheses were calculated from data of the highest resolution shell.

[b] $R_{merge} = \Sigma_{hkl}\Sigma_i | I_i(hkl) - \langle I(hkl)\rangle | / \Sigma_{hkl}\Sigma_i I_i(hkl)$

[c] $R_{meas} = \Sigma_{hkl}\{N(hkl)/[N(hkl) - 1]\}^{1/2} \Sigma_i | I_i(hkl) - \langle I(hkl)\rangle | / \Sigma_{hkl}\Sigma_i I_i(hkl)$

[d] $R_{pim} = \Sigma_{hkl}\{1/[N(hkl) - 1]\}^{1/2} \Sigma_i | I_i(hkl) - \langle I(hkl)\rangle | / \Sigma_{hkl}\Sigma_i I_i(hkl)$

[e] Correlation coefficient between intensities from random half-data sets.

[f] $R_{work} = \Sigma| |F_{obs}|-|F_{calc}| | / \Sigma|F_{obs}|$, where F_{calc} and F_{obs} are the calculated and observed structure factor amplitudes, respectively.

[g] R_{free} = as for R_{work}, except that 5% of the total reflections were selected at random and omitted from refinement.

[h] Calculated using MolProbity (http://molprobity.biochem.duke.edu).
